# Initial resuscitation of hemorrhagic shock

**DOI:** 10.1186/1749-7922-1-14

**Published:** 2006-04-27

**Authors:** Michael M Krausz

**Affiliations:** 1 Department of Surgery A, Rambam Medical Center, and the Technion-Israel Institute of Technology, P.O.B 9602, Haifa 31096, Israel

## Abstract

The primary treatment of hemorrhagic shock is control of the source of bleeding as soon as possible and fluid replacement. In controlled hemorrhagic shock (CHS) where the source of bleeding has been occluded fluid replacement is aimed toward normalization of hemodynamic parameters. In uncontrolled hemorrhagic shock (UCHS) in which bleeding has temporarily stopped because of hypotension, vasoconstriction, and clot formation, fluid treatment is aimed at restoration of radial pulse, or restoration of sensorium or obtaining a blood pressure of 80 mmHg by aliquots of 250 ml of lactated Ringer's solution (hypotensive resuscitation). When evacuation time is shorter than one hour (usually urban trauma) immediate evacuation to a surgical facility is indicated after airway and breathing (A, B) have been secured ("scoop and run"). Precious time is not wasted by introducing an intravenous line. When expected evacuation time exceeds one hour an intravenous line is introduced and fluid treatment started before evacuation.

Crystalloid solutions and blood transfusion are the mainstays of pre-hospital and in-hospital treatment of hemorrhagic shock. In the pre-hospital setting four types of fluid are presently recommended: crystalloid solutions, colloid solutions, hypertonic saline and oxygen-carrying blood substitutes. In unstable or unresponsive hemorrhagic shock surgical treatment is mandatory as soon as possible to control the source of bleeding.

Hemorrhagic shock is defined as a condition of reduced perfusion of vital organs leading to inadequate delivery of oxygen and nutrients necessary for normal tissue and cellular function. The understanding of the pathophysiology of shock has made significant progress only in the late 19^th ^and early 20^th ^centuries. Claude Bernard suggested that the organism attempts to maintain constancy of its *milieu interie *despite external forces that attempt to disrupt it [1].

Walter B. Cannon introduced the term *homeostasis *to describe the equilibrium maintained in the internal environment, and is credited for the first proposal to cause deliberate hypotension in order to reduce internal hemorrhage in uncontrolled hemorrhage before control of bleeding vessels [2].

Alfred Blalock proposed in 1934 four categories of shock: hypovolemic, vasogenic (septic), cardiogenic and neurogenic shock [3]. Hypovolemic shock the most common type results from loss of circulating blood volume due to loss of whole blood (hemorrhagic shock), plasma, interstitial fluid or a combination.

In 1947 Wiggers developed an animal model of graded controlled hemorrhagic shock by uptake of shed blood into a reservoir to maintain a predetermined level of hypotension [4].

This classic model was used by G. Tom Shires in the 1960s and 1970s to demonstrate that a large extracellular fluid (ECF) deficit occurred in prolonged severe hemorrhagic shock which was greater than could be attributed to vascular refill alone [5]. Only the infusion of both shed blood and lactated Ringer's solution to replace the ECF deficit, replaced the red cell mass, plasma volume, and ECF. Based on this data, the advocates of early aggressive resuscitation argued that the need for increasing cardiac output and oxygen delivery to maintain microvascular perfusion and oxygenation, exceeds any risk of accentuating hemorrhage and therefore trauma victims in hypotensive hemorrhage should receive large volumes of fluids as early as possible.

Aggressive fluid resuscitation during the Vietnam War with red blood cells, plasma, and crystalloid solutions allowed patients who previously would have succumbed to hemorrhagic shock to survive. Renal failure became a less frequent clinical problem, vital organ function was better sustained, but fulminant pulmonary failure termed "DaNang lung" or "Acute Respiratory Distress Syndrome (ARDS)" appeared as an early cause of death after severe hemorrhage.

Additional studies by this group demonstrated that this prolonged period of hemorrhagic hypotension was associated with the development of microvascular injury with marked ECF deficit which could be corrected only by the administration of isotonic crystalloids in volumes 2 to 3 times the estimated blood loss to achieve survival. This was the basis of the current well known dogma "3 to 1 rule" for the treatment of hemorrhagic shock, which was adopted by the ATLS for the treatment of trauma casualties [6]. It was recommended that the early treatment of hemorrhagic shock includes primarily the control of external bleeding and early intravenous administration of 2000 ml of crystalloids through a large bore-hole catheter.

This practice however, has recently been challenged in clinical trials [7,8] and experimental animal models [9-12] of uncontrolled hemorrhagic shock. It was observed that attempting to increase blood pressure to normal by aggressive fluid resuscitation in uncontrolled hemorrhagic shock resulted in increased bleeding from injured vessels, hemodynamic decompensation, and increased mortality, when compared to no fluid resuscitation [8,13,14] or hypotensive resuscitation (permissive hypotension) [15,16].

This fundamental dissimilarity in the hemodynamic response between controlled hemorrhagic shock (CHS) in which the bleeding source has been occluded, and uncontrolled hemorrhagic shock (UCHS) in which bleeding has temporarily stopped because of hypotension, vasoconstriction, and thrombus formation, constitutes the basis for our guidelines of fluid resuscitation in civilian as well as military trauma [17].

In CHS where the external bleeding source has been occluded as well as in UCHS where bleeding has temporarily stopped due to hypotension, when evacuation time is estimated to be shorter than one hour (usually urban trauma), immediate evacuation to a surgical facility is indicated after the airway and breathing (A, B) have been secured ("scoop and run"). Precious time is not wasted by introducing an intravenous line before evacuation, but infusion can be started en route to the medical facility.

When evacuation time is expected to be longer than one hour, an intravenous line is introduced and fluid resuscitation is started before evacuation.

Fluid resuscitation in CHS is aimed toward normalization of hemodynamic parameters, in contrast to UCHS, where hemostasis cannot be safely achieved, and early rapid evacuation to a surgical facility is considered the most important step of management after the airway and breathing have been secured. If transportation means are readily available fluid treatment can be started during evacuation.

According to the guidelines of the Israeli Defense Forces (IDF) fluid resuscitation of CHS is aimed toward normalization of hemodynamic parameters, in contrast to UCHS where the principles of hypotensive resuscitation are operative and treatment is started when one of the three parameters is documented [18]:

a. Altered sensorium

b. Radial pulse cannot be palpated

c. Systolic blood pressure dropped below 80 mmHg.

Fluid treatment does not include the automatic early administration of 2000 ml of lactated Ringer's solution as recommended by the ATLS guidelines, but repeated aliquots of 250 ml are administered with continuous monitoring, aiming at a systolic blood pressure of 80 mmHg, appearance of a radial pulse, or regained consciousness. In several studies it has been demonstrated that the prognosis of brain injuries is primarily dependent on cerebral perfusion [19]. Therefore, it was recommended that in central nervous system injuries with hemorrhagic shock fluid treatment is aimed toward a systolic blood pressure of 100 mmHg.

Aggressive fluid infusion to achieve normal hemodynamic parameters is prohibited in UCHS because it may renew internal bleeding ("pop the clot"). Massive fluid resuscitation is withheld until the time of the surgical intervention. When the expected transportation time to the medical facility exceeds one hour, hemodynamic evaluation is repeated every 15 minutes, and if systolic blood pressure drops below 80 mmHg, radial pulse cannot be palpated or the sensorium deteriorates, aliquots of 250 ml of Ringer's lactate solution are infused in order to maintain this blood pressure.

## Type of infused fluids

Infusions of crystalloid solutions and blood transfusions are the mainstays for the pre-hospital and in-hospital treatment of severe hemorrhagic shock. Blood is required for repletion of oxygen-carrying capacity, but it is usually not readily available in the prehospital settings because it requires refrigeration and typing. In the pre-hospital setting there are 4 types of fluids that are presently recommended for treatment of hemorrhagic shock:

### 1. Crystalloids

Lactated Ringer's solution is the most widely available and frequently used balanced salt solution for fluid resuscitation in hemorrhagic shock. It is safe and inexpensive, and it equilibrates rapidly throughout the extracellular compartment, restoring the extracellular fluid deficit associated with blood loss. Because of the rapid equilibration of balanced salt solutions into the extracellular space, larger volumes may be required for adequate resuscitation, resulting in decreased intravascular oncotic pressure. Although the use of crystalloids has been routine for resuscitation of patients with acute blood loss, several studies have raised questions regarding the effects of resuscitation regimens on aspects of the immune response to hemorrhagic shock. It was observed by Rhee et al. [20] that lactated Ringer's solution exacerbated neutrophil superoxide burst activity and increased neutrophil adherence. Also, it has been shown that aggressive crystalloid resuscitation was followed by increased cytokine activation including IL-1, IL-6, and TNF. [21]. The significant advantage of the currently available lactated Ringer's solution is that it provides a source of bicarbonate as a result of the metabolism of lactate to CO2 and H2O; and unlike bicarbonate, lactated Ringer's solution does not precipitate calcium when it is added to intravenous fluids.

### 2. Colloid solutions

The use of colloid solutions that tend to remain in the intravascular compartment has been advocated for treatment of hemorrhagic shock. Several colloid solutions were studied in clinical practice including human albumin, hydroxyl ethyl starch (HES), and dextran. Because colloid solutions remain briefly in the intravascular compartment, a lower total volume of resuscitative fluid is required to attain hemodynamic stability compared to crystalloid solutions. However, colloid solutions are more expensive, may bind and decrease serum ionized calcium, decrease circulating levels of immunoglobulines, and may further compromise the extracellular fluid volume deficit rather than restoring it. Numerous experimental and clinical studies have compared crystalloid and colloid fluid resuscitation [22,23]. There is no clinical evidence that appropriate resuscitation with balanced salt solution is associated with any harmful effects on pulmonary function when guided by hemodynamic parameters [24]. No protective effect of colloid solutions on post-resuscitation pulmonary function was demonstrated, even though colloid solutions do produce transiently greater intravascular expansion per unit compared to crystalloid solutions. Colloid solutions are recommended in military scenarios because of a major concern with regard to resuscitation of hemorrhage in the military setting where considerable weight and volume of crystalloid solutions must be transported in the field sometimes on the back of the medical professionals. This results in an inadequate bulk of the Ringer's lactate solution that is transported to the frontline and thus compromises the resuscitation phase in forward areas of deployment. In addition, patients in hemorrhagic shock in a combat area are frequently dehydrated, presenting an additional problem for successful resuscitation.

### 3. Hypertonic solutions

Clinical and experimental studies have demonstrated that a small volume of hypertonic saline (5 ml/kg NaCl 7.5%) with or without dextran can be an effective initial resuscitation solution. Hypertonic solutions improve microvascular flow, control intracranial pressure, stabilize arterial pressure and cardiac output with small-volume infusion, with no deleterious effects on immune functions [25-27]. Based on the safety and efficacy of hypertonic saline, with the need for simplicity, limited volume that can be carried in the field particularly in military scenarios, and the relative low cost, the Committee on Fluid Resuscitation for Combat Casualties of the Institute of Medicine [28] concluded that the initial fluid resuscitation of the hemorrhaging battlefield casualty should be a 250 ml bolus of 7.5% saline delivered by a rapid infusion system. Systemic access would be achieved via an intraosseous needle or by intravenous access. This practice, however, has been recently challenged in clinical trials as well as laboratory studies of UCHS. Meta-analysis of clinical studies of hypertonic saline treatment of traumatic hemorrhagic shock showed an increase in blood pressure and cardiac output but there was no significant improvement in survival [24]. In animal studies hypertonic saline treatment of UCHS secondary to large-vessel injury resulted in increased bleeding from injured blood vessels, hemodynamic decompensation and increased mortality [9-11]. In UCHS secondary to solid organ injury (massive splenic injury) hypertonic saline infusion improved hemodynamics but did not increase bleeding from the injured solid organ [12].

### 4. Oxygen-carrying blood substitutes

hold promise as effective resuscitation fluids that may improve oxygen carrying capacity without problems of storage, compatibility, and disease transmission that are associated with standard blood transfusion. Oxygen-carrying blood substitutes can be generally divided into two types: fluorocarbon-based synthetic oxygen carriers and stroma-free cross linked human or non-human hemoglobin products. The fluorocarbon emulsions are easy to produce, have a long shelf life, and have minimal infectious or immunogenic effects. Potential disadvantages include the requirement of a high FiO2, and rapid plasma clearance. Hemoglobin-based oxygen carriers are notable for high oxygen carrying capacity, an appreciable oncotic effect, and prolonged shelf life. Disadvantages include short plasma half life, potential renal toxicity, hypertensive effects, and the potential of immunogenic effects. Further clinical trials to establish the optimal dosage, efficacy, safety, and the effect on outcome are indicated before oxygen-carrying blood substitutes are implemented in routine clinical practice.

Once oxygenation and circulating volume have been restored, re-evaluation of the clinical situation is in order. Vital signs, mental status, urinary output, and capillary refill should be assessed regularly throughout the resuscitation. Initiation of central monitoring may be indicated at this time, if the response to initial resuscitation has been less than expected, or if blood loss is ongoing. Blood should be drawn to assess hematological, coagulation, electrolyte and metabolic status. Electrolyte and metabolic disorders as well as coagulation deficiencies should be corrected. Arterial blood gases should be obtained to determine the adequacy of oxygenation. Management of alternations in oxygenation, ventilation, pH, fluid and electrolyte balance should now be based on clinical evaluation and laboratory measurement. Blood components may also be used at this stage to replace identified deficiencies.

Most cases of unresponsive hemorrhagic shock to fluid management in the trauma patient are due to ongoing losses of blood volume or myocardial dysfunction. While initial stabilization is taking place, attention should be directed to prompt arrest of bleeding. Aggressive restoration of normal blood pressure without arrest of internal hemorrhage will enhance further losses of blood volume by increasing flow and impeding coagulation at the site of injury. Mild to moderate hypotension allows for clot formation and slows bleeding from injured blood vessels (hypotensive resuscitation). The hemodynamically unstable injured victim should be brought to surgery as soon as possible and the source of bleeding promptly identified and arrested.
